# Evaluation of quality of life in endometriosis patients before and after surgical treatment using the EHP30 questionnaire

**DOI:** 10.1186/s12905-022-02111-3

**Published:** 2022-12-22

**Authors:** D. Tiringer, A. S. Pedrini, M. Gstoettner, H. Husslein, L. Kuessel, A. Perricos, R. Wenzl

**Affiliations:** 1 Department of Obstetrics and Gynecology, Medical University of Vienna, Waehringer Guertel 18-20, 1090 Vienna, Austria; 2 Department of Internal Medicine I, Medical University of Innsbruck, Innsbruck, Austria

**Keywords:** Endometriosis, Quality of life, EHP-30 questionnaire, Surgical therapy, Laparoscopic surgery

## Abstract

**Backround:**

Endometriosis is one of the most common gynecological illnesses causing extensive psychological, physical and social impact on patient’s life and exerts negative effects on health-related quality of Life (HRQoL). However, the effects of surgery on the postoperative HRQoL in the different endometriosis subgroups have not been fully evaluated.

**Methods:**

We performed a comparative retrospective study between 2014 and 2018 at the Medical University of Vienna, including all patients with surgically confirmed endometriosis who had completed the standardized Endometriosis Health Profile-30 (EHP-30) questionnaire 1 day after surgery (the questions refer to the 4 weeks preoperatively) and 6–10 weeks postoperatively.

**Results:**

Compared to preoperative values, we found significant benefits, regarding postoperative conditions, in our study group (n = 115) in all five categories, “pain” (HR 0.78, *p* < 0.001); “self-determination” (HR 0.92, *p* < 0.001); “emotional health” (HR 0.83, *p* < 0.001);” social environment” (HR 0.67, *p* < 0.001); and “self-image” (HR 0.47, *p* < 0.001). Patients with only peritoneal endometriosis had the lowest preoperative clinical symptoms and there were no significant changes in any of the categories. In the subgroups deep infiltrating endometriosis (DIE) and DIE + ovarian endometrioma, surgical intervention results in a significantly greater improvement in all categories of EHP 30 compared to ovarian endometrioma without DIE or peritoneal endometriosis.

**Conclusion:**

Our study shows, that especially women with DIE—with or without ovarian endometrioma—demonstrate a more pronounced benefit from surgical therapy compared to patients with peritoneal endometriosis or endometrioma without DIE.

## Backround

Endometriosis, defined as the presence of endometrium-like tissue outside the uterine cavity, is a chronic disease affecting women in their reproductive age [1, 2]. One of the main symptoms reported by patients is pain that can be expressed in a variety of symptoms, including dysmenorrhea, dyspareunia, and chronic pelvic pain [3]. These symptoms have an adverse impact on social, mental and physical wellbeing. Additionally, the impairment of HRQoL can significantly affect professional and private relationships, sexuality, social contacts, family planning (due to infertility) or psychological well-being [4–6]. Recent studies confirmed that women with endometriosis have a lower HRQoL compared to the general population [2, 7, 8].

Therapy of endometriosis comprises surgery, hormonal contraceptives or pain therapy. Still, little is known about the quantitative impact of surgery on the patients HRQoL. In the past few years, there has been increasing progress in the development and validation of psychometric questionnaires in order to asses HRQoL of endometriosis patients in clinical routine [9, 10]. Several studies on HRQoL in patients with endometriosis have been performed with conflicting results and using different questionnaires. In addition, only a few studies focused on HRQoL in relation to the different forms of endometriosis [11–13].

The 30-item Endometriosis Health Profile (EHP-30) developed by Georgina Jones, is a specific HRQoL scale derived from interviews of patients with endometriosis [14–16]. The EHP-30 is the best validated disease-specific questionnaire for the documentation of endometriosis related impact on patients´ life. This questionnaire is sensitive to changes and is thus a suitable tool to evaluate treatment effects on the health status of patients with endometriosis. The EHP-30 consists of a 30-item core questionnaire applicable to all women with endometriosis, categorized into five subscales—pain (11 items), control and powerlessness (6 items), emotions (6 items), social support (4 items) and self-image (3 items). In addition, the EHP-30 also consists also of a modular part, which does not apply to all women, including questions regarding work, relationship with children, sexual relationship, feelings about the medical profession, feelings about treatment and feelings about infertility [17]. The question remains if our therapeutic interventions help to improve those impairments. Thus, the aim of this study was to determine if surgical therapy of endometriotic lesions results in an improvement of HRQoL in relation to the different forms of endometriosis.

## Methods

### Patients

**Fig. 1:**
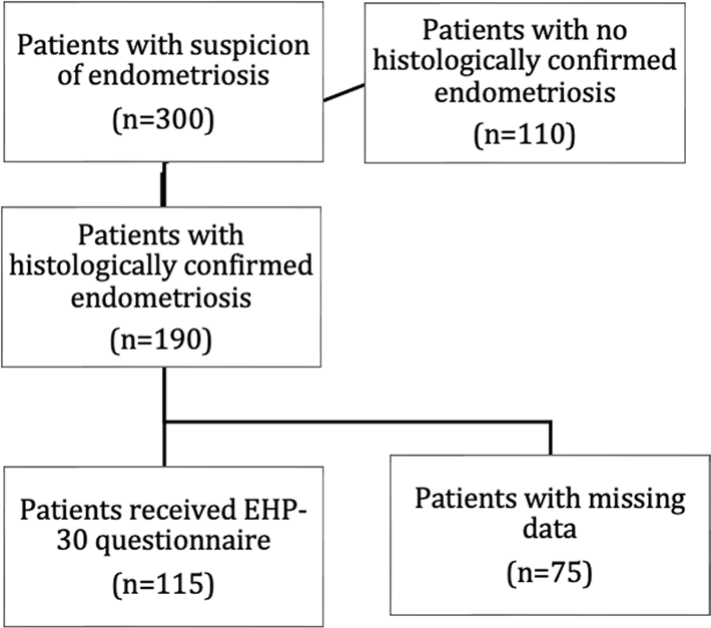
Flow chart of patients’ inclusion

We included all consecutive patients operated due to suspected endometriosis at the Medical University of Vienna, Austria, between 2014 and 2018, who gave their written informed consent to participate in our study. Inclusion criteria comprised age 18–50 years, histological confirmation of endometriosis and ability to complete the EHP-30 questionnaire. Women with a current malignancy defined as < 10 years after breast cancer or < 5 years after other malignant tumors, were excluded. Additionally, excluded were patients with infections such as HIV, Hepatitis (A, B, C), tuberculosis, and systemic autoimmune diseases. The respective patient flow-chart is shown in Fig. 1. The study protocol was approved by the local ethic committee (EK code 1145/2018).

### Classification of endometriosis

Based on the surgical report, patients with histologically confirmed endometriosis were categorized based on the rASRM and ENZIAN [18, 19] classification, retrospectively. Patients were then divided into four groups; group 1. peritoneal endometriosis; group 2. ovarian endometrioma; group 3. deep infiltrating endometriosis (DIE); and group 4. DIE + ovarian endometrioma [20]. As peritoneal endometriosis we defined all superficial (subperitoneal invasion < 5 mm) peritoneal foci located in the abdomen. All endometriotic ovarian cysts and all foci ≥ 5 mm infiltrating the ovarian surface were considered as ovarian endometriosis. All lesions infiltration the subperitoneal stroma ≥ 5 mm were classified as DIE.

### Surgical intervention

Laparoscopic surgery was performed at the general hospital of Vienna in all participating patients. While the intraoperative steps were decided individually, the overall result was the excision of all endometriotic lesions in the abdominal cavity in all patients. All endometriomas were conservatively removed, in cases of DIE of more than 3 cm diameter of the bowel, segmental resection was performed. All other intestinal lesions were shaved.

### EHP questionnaire

The first EHP-30 questionnaire was filled out 1 day before surgery. The questions referred to the past 4 weeks preoperatively. Six to ten weeks postoperatively, our patients filled out the questionnaire a second time to evaluate the postoperative quality of life. As the modular part did not apply to all patients, we did not include it in our analysis.

### Statistics

All EHP questionnaires were entered in SciCoMed, exported as an Excel table and then imported into IBM-SPSS. The data was evaluated anonymized. The data of the EHP-30 questionnaire were generated according to the algorithm developed by Jones et al. evaluated in 2001 [14]. Response categories are rated on a five-point scale (0–4). Raw scores (the sum of items in each subscale) are translated into a score (each raw score is first divided by the maximum possible raw score and multiplied by 100) ranging from 0 (best possible health status) to 100 (worst possible health status). The maximum value of 100 corresponds to that of the maximum load and 0 means no impairment.

After testing for normal distribution using Shapiro Wilk, all demographic data were given a Q–Q plot (quantile–quantile plot) in a frequency table. Depending on the scaling, the clinical data and categorical variables were given with absolute and relative frequency or by mean and standard deviation (SD). Correlations between socio-demographic and clinical data (age, partnership status, BMI, menarche, menstrual cycle, bleeding intensity, bleeding duration) and the parameters of the EHP-30 were determined. *p*-values < 0.05 were considered statistically significant. All statistical analyses were performed with IBM SPSS software version (Vienna/Austria).

## Results

### Demographics

Initially, 300 patients suspected of having endometriosis and a planned surgery at the Medical University of Vienna, were screened. Of these, 190 patients had histologically confirmed endometriosis and met the relevant inclusion and exclusion criteria and gave their consent to participate in our study. Due to missing postoperative data, 75 of these patients had to be excluded. Patient´s characteristics and localization of endometriosis are shown in Table 1.

**Table 1:** Patient characteristics and localization of endometriosis

	*n* (%)
Age diagnosis (years) (mean ± SD)	32 ± 7
BMI (mean ± SD)	23.2 ± 4.4
Partnership	
Single	28 (24.3)
In a partnership	87 (75.7)
Pregnancies	
0	71 (61.7)
1	28 (24.3)
2	9 (7.8)
> 2	7 (6.1)
Births	
0	87 (75.7)
1	21 (18.3)
2	6 (5.2)
> 2	1 (0.9)
Smoker	
Smoker	27 (23.5)
Non-smoker	88 (76.5)
Common symptoms (mulptiple selections possible)	
Dysmenorrhea	108 (93.9)
Dyspareunia	93 (80.9)
Dysuria	75 (65.2)
Abdominal pain	62 (53.9)
Chest pain	12 (10.4)
Menarche (age)	
Mean (± SD) 12.7 ± 1.7	
8–10 years	7 (6.1)
11–14 years	94 (81.7)
15–17 years	14 (12.2)
Bleeding duration (days)	
Mean (± SD) 5.0 ± 2.5	
1–4 days	51 (44.3)
5–8 days	57 (49.6)
9–14 days	7 (6.1)
Bleeding intensity	
Amenorrhea	5 (4.3)
Light	7 (6.1)
Middle	43 (37.4)
Strong	60 (52.2)
Hormonal therapy in the last 3 months	
Yes	27 (23.5)
Combined hormonal therapy	9 (33.3)
Progesterone only	18 (66.7)
No	83 (72.2)
Not specified	5 (4.3)
Period of time from the beginning of pain until diagnosis (years)	
Mean (± SD) 4.8 ± 6.1	
Present wish to have children	
Yes	41 (35.7)
No	74 (64.3)
Peritoneal endometriosis	26 (22.6)
Ovarian endometriosis	23 (20%)
DIE	52 (45.2)
DIE + ovarian endometriosis	14 (12.2)

The table should be placed in the results part after the section *Demographics*

### EHP-30

Out of the core and modular questionnaire all categories were analyzed in detail in the total population as well as in the 4 subgroups.

### Impact of patient characteristics on EHP-30

A lower BMI was positively associated with “emotional health” (rs = 0.251, *p* = 0.007) and “self-image” (rs = 0.245, *p* = 0.008). There was a significant negative correlation between partnership status and the emotional health category (rs = − 0.191, *p* = 0.041). Patients in a partnership had less negative impact on emotional health status. No significant correlations between age and such as emotional health or pain sensitivity were recorded.

### Impact of endometriosis on EHP-30

Additionally, the EHP-30 categories were compared between the four endometriosis subgroups. The highest pre-operative pain levels were observed in patients DIE and DIE + ovarian endometrioma (42.2 ± 22.1). The most significant improvement in respect to EHP-30 was also seen in these categories (− 27.8 ± 6.8).

### Impact of surgery on the EHP-30

**Fig. 2:**
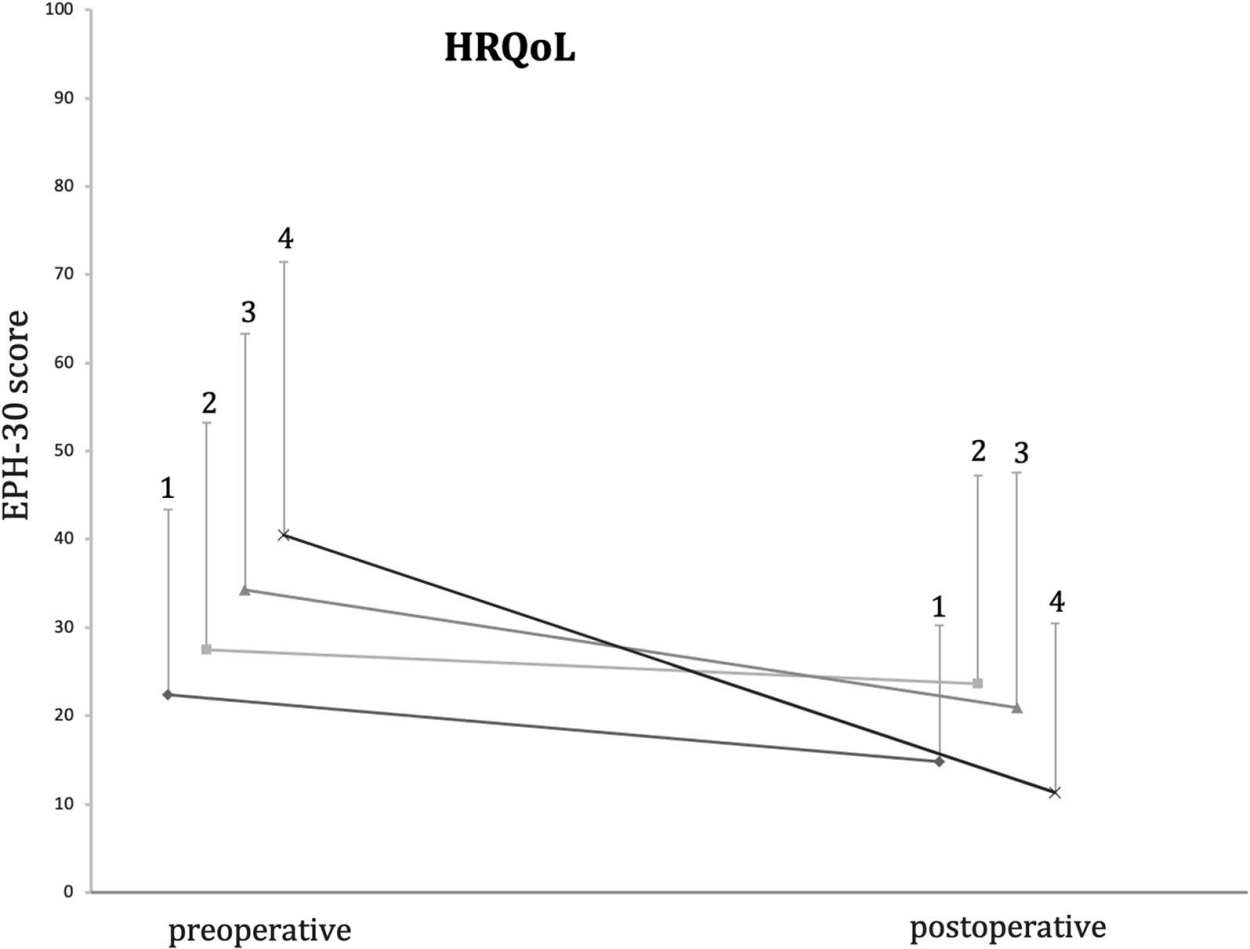
Comparison of pre- and postoperative Health related quality of Life (HRQoL) values in the four endometriosis groups. (1) patients with peritoneal endometriosis; (2) patients with ovarian endometriosis; (3) patients with deep infiltrating endometriosis; (4) patients with deep infiltrating endometriosis + ovarian endometrioma

**Fig. 3:**
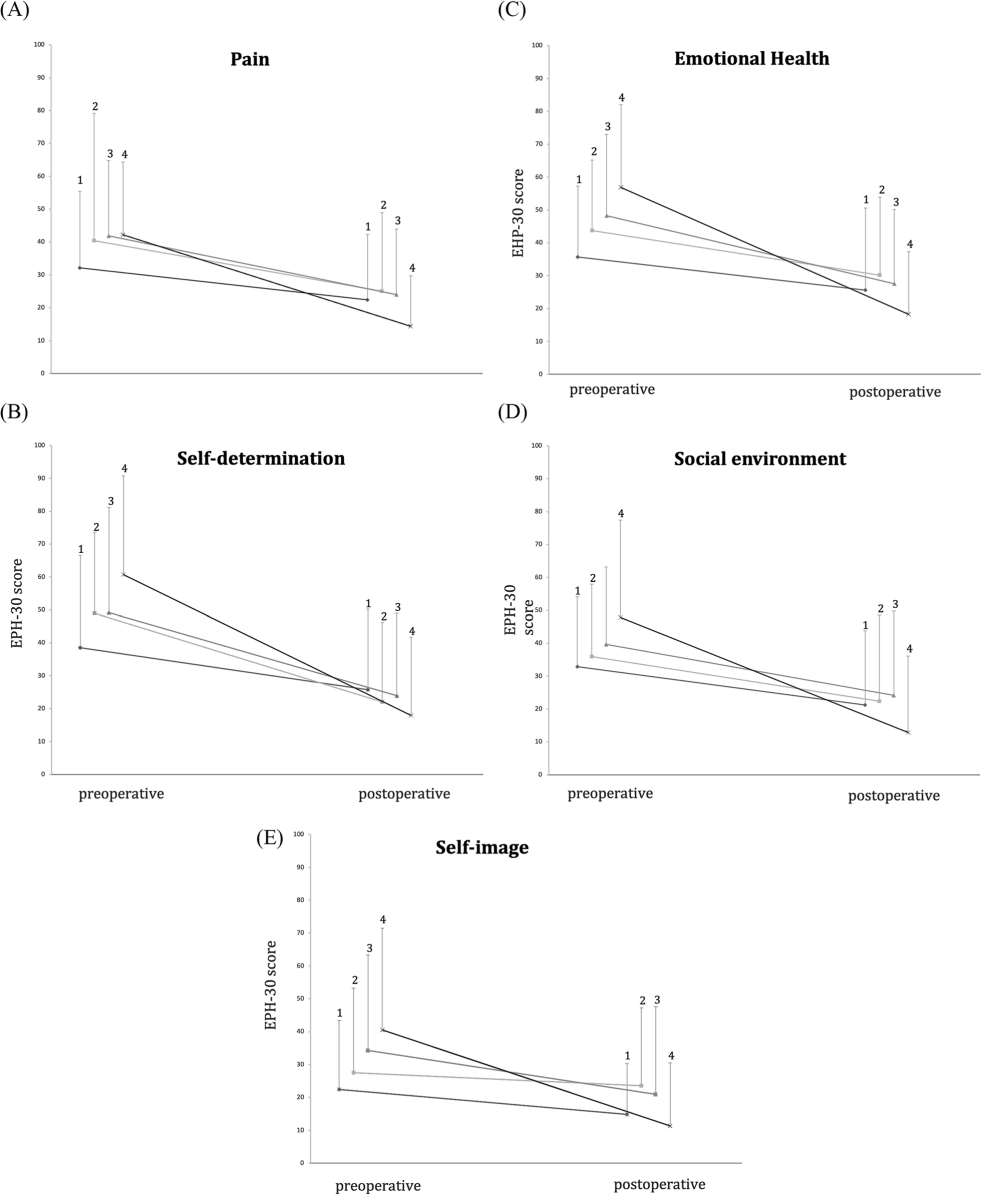
Changes of EHP-30 scores in the four endometriosis groups during the study period. Values are mean + pos SD shown by vertical bars. EHP subdomains scores range from 0 to 100. Lower score indicates fewer negative symptoms. (1) patients with peritoneal endometriosis; (2) patients with ovarian endometriosis; (3) patients with deep infiltrating endometriosis; (4) patients with deep infiltrating endometriosis + ovarian endometrioma. **A** Pain scores. **B** Self-determination scores. **C** Emotional-health scores. **D** Social environment scores. **E** Self-image scores. EHP-30, endometriosis health profile-30. *p < 0.05

**Fig. 4:**
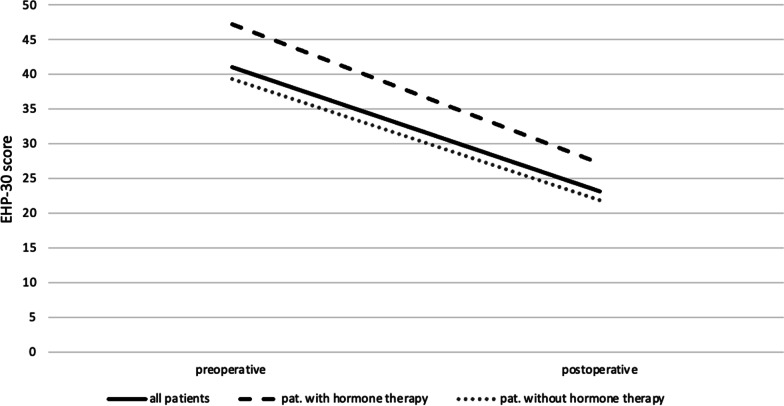
Changes of EHP-30 scores in patients under hormone therapy and in patients without intake of hormone therapy

All of the five main categories show a positive change in the quality of life postoperatively (Figs. 2 and 3): pain (HR 0.78, *p* < 0.001); self-determination (HR 0.92, *p* < 0.001); emotional health (HR 0.83, *p* < 0.001); social environment (HR 0.67, *p* < 0.001); and self-image (HR 0.47, *p* < 0,001). Furthermore, a positive change was seen in patients who were under hormone therapy as well as in patients who were not (Fig. 4).

When analyzing the five categories regarding the four endometriosis subgroups separately, we found the most significant improvements in the groups of DIE and DIE + ovarian endometrioma. In the group of ovarian endometrioma there was a significant improvement postoperatively in the categories “pain”, “self-determination” and “emotional health”. In the patients with peritoneal endometriosis, there were no significant changes in any of the five categories.

Aim of this study was to evaluate the effect of surgery on the HRQoL of patients with endometriosis. Using the EHP-30 questionnaire in the whole study group, we determined that all of the five main categories show a positive change and thus improvement of quality of life after laparoscopic surgery. HRQoL changes were also calculated separately in the four subpopulations (peritoneal, ovarian, DIE and DIE + ovarian endometrioma). Significant improvements were seen in the categories “pain”, “self-determination” and “emotional health” in all subgroups, except in the group of patients with peritoneal endometriosis.

In the categories “social environment” and “self-image”, there was only a significant change in the groups of DIE and DIE + ovarian endometriosis. We observed, that patients with only peritoneal endometriosis had the lowest preoperative clinical symptoms. Due to the more pronounced preoperative clinical symptoms particularly in DIE and DIE + ovarian endometrioma, surgical improvement seems to result in a greater change in the score and consecutively a greater improvement in the quality of life. Thus, the results of this study highlight, that especially patients with DIE and DIE + ovarian endometrioma benefit from a surgical intervention. A limitation of this study is the low patient number. Studies with larger subpopulations are thus required to validate our findings.

## Discussion

In clinical practice, routine evaluation of HRQoL in women who suffer from endometriosis is essential, both for the health-care provider and the patient [21]. In this study, longitudinal data of quality of life was obtained from a total of 115 patients with endometriosis. The median age of the recruited patients was 33 years, comparable to similar studies reporting an average age of 34 years [22–24]. Most of the included patients were in a partnership, a factor known to increase the probability of consulting a doctor because of painful intercourse or the desire to have children [13]. There was no correlation between age and “emotional health” (rs = − 0.154 *p* = 0.101). In contrast to another similar Austrian study, in which more advanced age led to a deterioration in “emotional health” [13]. A negative correlation between the BMI and “emotional health” or “self-image” was recorded, which means that a higher BMI led to a deterioration in emotional health and self-image. This finding has also been confirmed by another study [10].

Our data demonstrates that this disease affects many aspects of the quality of life of endometriosis patients. This finding has also been confirmed by other studies [10].

In the overall study population, a significant improvement was seen in all aspects of quality of life, excluding “feelings about the medical profession.” The exception of the last mentioned category may be due to the fact that the relationship with physicians is not necessarily related to the current health status. This was also reported in a study by Van de Burgt et al. 2013 [17].

In our patient collective, the strongest improvement after surgery was seen in the category “self-determination”, followed by the categories “pain” and “emotional health”.

Our data is comparable to the study by Jones et al. published in 2004, as the greatest positive change in the total population was revealed in the aspect of “self-determination” [22].

Comparing the subpopulations (peritoneal, ovarian, deep infiltrating endometriosis and TIE + ovarian endometriosis) amongst each other regarding all categories of EHP-30, differences could be recorded: “Pain”, “Self-determination” and “Emotional health” showed significant improvements in all subpopulations except in the group of patients with peritoneal endometriosis. In the categories “social environment” and “self-image”, only the deep infiltrating endometriosis and TIE + ovarian endometriosis groups showed a significant improvement.

One of the strengths of our study is that in all patients who presented with macroscopic endometriotic lesions during surgery, the stage of the disease was categorized. As larger lesions were excised and histologically evaluated, we histopathologically confirmed the diagnosis in all cases. All surgeries were performed by one of five senior members of the certified endometriosis center, who all used to same procedures. In all cases a residual free (R0) resection was achieved. In another study by Khong et al. patients with merely suspected endometriosis due to pelvic pain or infertility were included in an EHP-30 questionnaire study [16]. Furthermore, one additional strength lies in the preoperative and postoperative collection of the data, which was not carried out in other studies [13]. In addition, our collective is part of a prospective cohort design of well characterized endometriosis patients [25].

While the intake of a hormonal medication may in itself influence the QOL, we decided not to exclude these patients from our collective. Patients suffering from endometriosis often do not wish to interrupt hormonal treatment before surgery and we aimed to present a real-life patient collective consulting a tertiary endometriosis referral center.

However, due to the short follow-up period (6–10 weeks), no statement about the long-term effect can be given. In this regard, further studies are needed to assess the effectiveness of an operative treatment over a longer period. It should also be noted, that the questionnaires are always answered from a subjective perspective. Since the content validity of the EHP-30 is high, the results based on the questionnaire can be regarded as relevant despite the subjective answers. Nevertheless, it should be noted that many patients have been living with impairments in HRQoL such as pain for years and in some cases have learned to deal with its draw backs.

Confirming previous data [17, 22, 26], our study highlights that the EHP-30 can be regarded as a reliable instrument that reacts sensitively to changes. The preoperative and postoperative values can be used to determine the individual effect of surgical therapy regarding different types of endometriosis.

## Conclusion

The EHP-30 questionnaire shows a good overall performance in measuring HRQoL. The present work underlines, that the surgical treatment of endometriosis has a positive effect on all well-being parameters measured by the EHP-30. Significant improvement of EHP-30 was achieved in all endometriosis groups, except peritoneal endometriosis. Especially women with DIE—with or without ovarian endometrioma -show a pronounced benefit from surgery compared to peritoneal and ovarian endometrioma without DIE.

## Data Availability

The datasets used and/or analyzed during the current study are available from the corresponding author on reasonable request.
